# Floppy capsule appearance during phacoemulsification with mannitol in eyes with angle closure glaucoma

**DOI:** 10.5935/0004-2749.20200105

**Published:** 2024-02-11

**Authors:** Fatih Özcura, Saadet Gültekin Irgat

**Affiliations:** 1 Department of Ophthalmology, Kutahya Health Sciences University School of Medicine, Kutahya, Turkey

Phacoemulsification in short eyes can be challenging. Surgical manipulation in a shallow anterior chamber with increased vitreous pressure tends to increase in- traoperative complications. Intravenous mannitol is often administered shortly before surgery to decrease vitreous pressure prophylactically. General anesthesia is preferred; if local anesthesia is chosen, peribulbar, or retrobulbar anesthesia should be avoided to prevent retrobulbar pressure. Topical anesthesia is preferable but good eyelid akinesia is critical to prevent eyelid pressure. Regarding intraoperative precautions, surgery can be performed under high infusion bottle height to deepen the anterior chamber or the active fluidics systems can be used. Limited pars plana vitrectomy is another precaution to increase anterior chamber depth (ACD)^(1-3)^.

We present an unusual undulation and flaccidity of the lens capsule called floppy capsule in response to irrigation fluid currents during phacoemulsification, in three eyes of two patients with angle closure glaucoma, where preoperative mannitol was used to enlarge ACD. To the best of our knowledge, floppy capsule has not been described in the literature. Clinically, it appears similar to the floppy iris; however, it is distinct from zonular weakness or dialysis.

A 67-year-old man was referred to our glaucoma unit with primary angle closure glaucoma. The visual acuity was 20/40 in the right eye with hand motion in the left eye. Intraocular pressure (IOP) was 16 mmHg (no medication) and 33 mmHg (with maximal medication) in the right and left eyes, respectively. The ocular examination revealed bilateral grade 2 nuclear cataract and shallow anterior chamber. We performed laser peripheral iridotomy for the left eye. ACD was 1.68 mm and 1.74 mm and anterior chamber angle (ACA) was 11.4° and 9.9° in the right and left eyes, respectively. Axial length (AL) was 20.74 mm in the right eye and 20.24 mm in the left eye.

**Figure 1:**
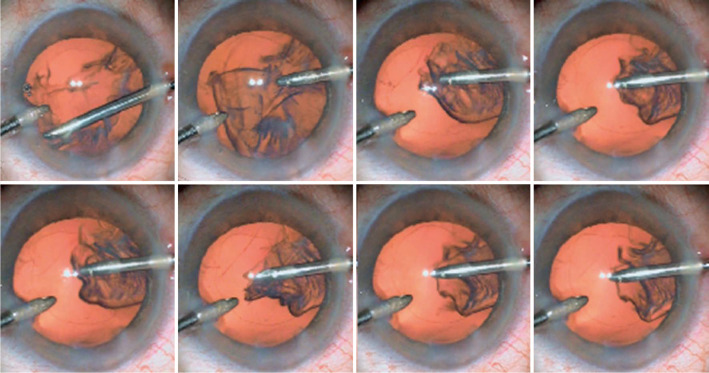
These serial photographs show floppy capsule movement during the irrigation or aspiration of the cortex.

We planned cataract extraction for both eyes of the patient with left priority. Intravenous mannitol 20% (1 g/kg) was administered one hour before surgery. We used topical anesthesia and eyelid akinesia with modi- fied van Lint techniques. We observed unusual undu- lation and flaccidity of the lens capsule in response to irrigation fluid currents during the irrigation or aspiration of the cortex (Figure 1).

IOP was 15 mmHg (no medication) and visual acuity increased to 20/30, 4 weeks after the surgery. Moreover, ACD increased to 4.71 mm and ACA widened to 30.2°. We performed right eye cataract surgery of the patient 2 months later using the same preoperative preparations, anesthesia, and surgical technique. Here too, we encountered unusual undulation and flaccidity of the lens capsule (Video 1).

**Video 1:**
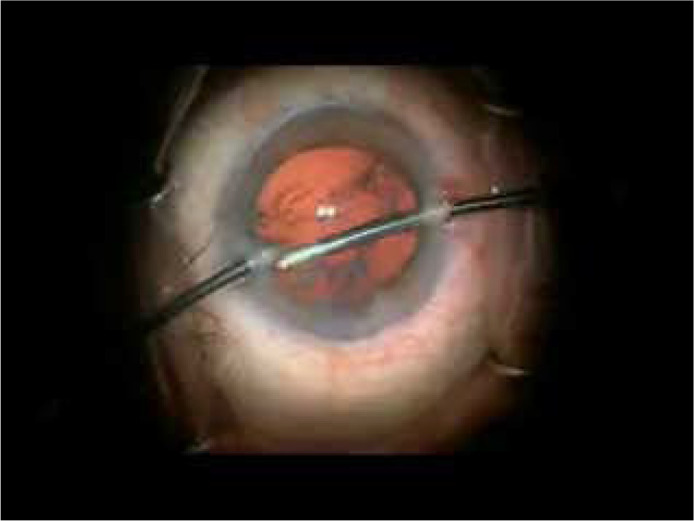
This video shows unusual undulation and flaccidity of lens capsule in response to irrigation fluid currents during surgery.

A 65-year-old man was referred to our glaucoma unit with uncontrolled angle closure glaucoma on his right eye. IOP was 45 mmHg despite IOP-lowering pharmacotherapy. The patients also had patent laser peripheral iridotomy. ACD, ACA, and AL were 2.12 mm, 15.1°, and 22.68 mm, respectively. Ocular examination revealed nuclear sclerosis and glaucomatous optic nerve damage. We performed lens extraction to reduce IOP using the same preoperative preparation, anesthesia, and surgical technique. We floppy movements of the lens capsule in response to irrigation fluid currents during surgery.

Mannitol is a hyperosmotic agent and increases osmotic pressure to drive fluid out of the extracellular space and into the vasculature. Animal and human studies have shown a significant reduction in vitreous volume after mannitol administration. The reduction in vitreous volume and separation of anterior hyaloid to posterior lens capsule facilitates the downward movement of the lens-iris diaphragm^(4)^. Moreover, the separation of the anterior hyaloid from the posterior lens capsule may increase the flexibility of the lens capsule. We believe that floppy movements can also be observed in other clinical scenarios (e.g., phacomorphic glaucoma or intumescent cataract) when using mannitol.

We have described an unusual undulation and fluctua tion of the lens capsule and have called it floppy capsule, inspired by floppy iris. These unusual movements of the lens capsule should not be considered as a zonular problem and surgery should continue as usual.
